# Opportunistic detection of anal intraepithelial neoplasia at colonoscopy

**DOI:** 10.1002/jgh3.12424

**Published:** 2020-10-14

**Authors:** Clara M Forbes, Jenny McCloskey, Geoffrey M Forbes

**Affiliations:** ^1^ School of Health and Medical Sciences University of Western Australia Perth Western Australia Australia; ^2^ Department of Sexual Health Royal Perth Hospital Perth Western Australia Australia

**Keywords:** anal intraepithelial neoplasia, colonoscopy, detection, human papilloma virus

## Abstract

**Background and Aim:**

Human papilloma virus‐associated anal intraepithelial neoplasia (AIN) precedes most anal cancers and can be detected at colonoscopy. We aimed to quantify AIN detection rates in a general population undergoing colonoscopy.

**Methods:**

A retrospective review of a community‐based practice for 2 years until December 2019 was conducted.

**Results:**

A total of 2525 patients (1051 males and 1474 females; median age 59 years) had 2608 colonoscopies. Ten patients (two males and eight females; median age 57.5 years) had incidentally detected AIN (condyloma acuminatum or AIN1, *n* = 4; AIN2 or 3, *n* = 6). AIN was detected in 1 of 261 (95% CI 1/142–1/480) colonoscopies and 1 of 163 (95% CI 1/83–1/321) colonoscopies in women over 40 years old.

**Conclusions:**

Opportunistically detecting AIN, especially in women over 40 years old, should be an important adjunct to colonoscopy‐based colorectal neoplasia detection.

## Graphical abstract

Human papilloma virus‐associated anal intraepithelial neoplasia (AIN) precedes most anal cancers and can be detected at colonoscopy. We aimed to quantify AIN detection rates in a general population undergoing colonoscopy. A retrospective review of a community‐based practice for 2 years until December 2019 was conducted. A total of 2525 patients (1051 males and 1474 females; median age 59 years) had 2608 colonoscopies. Ten patients (two males and eight females; median age 57.5 years) had incidentally detected AIN (condyloma acuminatum or AIN1, *n* = 4; AIN2 or 3, *n* = 6). AIN was detected in 1 of 261 (95% CI 1/142–1/480) colonoscopies and 1 of 163 (95% CI 1/83–1/321) colonoscopies in women over 40 years old. Opportunistically detecting AIN, especially in women over 40 years old, should be an important adjunct to colonoscopy‐based colorectal neoplasia detection.

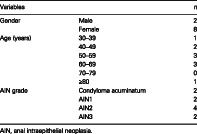

## Introduction

Human papillomavirus (HPV) infection is common, with a lifetime probability of infection greater than 80% for sexually active, nonvaccinated men and women.^1^ Infected individuals are at risk of serious sequelae, including anal, cervical, and oropharyngeal malignancy.^2^ Anal cancer is uncommon, with an age‐adjusted incidence in Australia of 1.6 persons per 100 000.^3^ However, the incidence of anal cancer in Australia has been increasing, particularly in women; in 1982, the incidence was 0.8 per 100 000 females, and this increased to 1.7 in 2015.^3^ Australian teenagers are being vaccinated against common strains of HPV in a nationwide program that commenced in 2007; this is anticipated to substantially reduce both cervical and anal cancer rates.^4^ However, those currently aged older than 30 years are often unvaccinated and at risk of HPV‐related disease.

Most anal cancers are of squamous cell and are preceded by anal intraepithelial neoplasia (AIN). Low‐grade squamous intraepithelial lesions (LSILs) comprise condyloma acuminatum and AIN1; high‐grade squamous intraepithelial lesions (HSILs) comprise AIN2 and AIN3.^5^ There are recognized populations that are at high risk for developing AIN, on which published data have concentrated. These include human immunodeficiency virus (HIV)‐positive subjects and those partaking in receptive anal intercourse.^6^ Previous cervical intraepithelial neoplasia (CIN) is a major risk factor for anal cancer in women.^7^, ^8^, ^9^, ^10^

Colorectal cancer screening and prevention has become an important part of population‐based health care. AIN is frequently asymptomatic and can only be accurately examined for endoscopically. Colonoscopy provides an opportunity to detect AIN in the general population, where the incidence of AIN has not been studied. We evaluated the detection rate and incidence of AIN in a community‐based colonoscopy practice, with a view to demonstrate the adjunctive benefit to colorectal neoplasia detection.

## Methods

This retrospective evaluation arises from a single‐operator, community‐based medical practice in Perth, Australia. Patients underwent colonoscopy after bowel preparation, including a 48‐h low‐fiber diet followed by oral lavage, utilizing polyethylene glycol to make 1 L and three packets of 10 mg of sodium picosulfate as a split‐dose regimen. Propofol‐based sedation was administered by an anesthetist. Anal canal epithelial and transition zone lesions were routinely examined for, and pinch biopsies were taken when AIN was suspected on endoscopic appearances. Specimens obtained were reviewed by specialist gastrointestinal pathologists. Patients with AIN were referred to a sexual health physician for local ablative therapies under high‐resolution anoscopy.^11^

Data were collected from all patients undergoing colonoscopy from 1 January 2018 to 31 December 2019. These data describe the population from which our cohort arose and provide associated quality assurance information. For patients with AIN, ages are presented in deciles, and the indication for colonoscopy is not given for reasons of patient anonymity; 95% confidence intervals for the rate of AIN detection at colonoscopy and 2‐year incidence rate were calculated using the Wilson score interval for small numerators.

When calculating median age, age deciles, and gender, patients with more than one colonoscopy in the period were only accounted for once. Cecal/terminal ileal intubation rate and adenoma and sessile serrated lesion detection rates (ADRs and SDRs) were calculated for all colonoscopies; ADRs and SDRs were also calculated as per the Gastroenterological Society of Australia (GESA) guidelines, where eligible patients are those 50 years and older, have intact colons, and do not have inflammatory bowel disease as the reason for colonoscopy.^12^ Clinical audit approval was obtained through the Western Australia Governance, Evidence, Knowledge and Outcome (GEKO) quality management system.

## Results

Over a 2‐year period, 2525 patients had 2608 colonoscopies. Demographic data for all patients, including gender, age decile, and indication for colonoscopy, are presented in Table 1. Overall, the median age was 59 years; 41.6% were male and 58.4% female.

**Table 1:** Demographic and quality assurance data for all patients undergoing colonoscopy

Variables		*n* or %
Gender	Male	1051
	Female	1474
Age (years)	≤19	27
	20–29	94
	30–39	220
	40–49	391
	50–59	543
	60–69	726
	70–79	471
	≥80	53
Indication for colonoscopy	Polyp surveillance, follow‐up colon cancer	633
	Altered bowel habit	401
	Positive FOBT/colorectal neoplasia screening	353
	Family history of colorectal cancer	325
	Bleeding	299
	Anemia and iron deficiency	228
	Bloating, pain	219
	Follow‐up colitis	75
	Follow‐up diverticulitis	32
	Abnormal imaging	26
	Other	17
Cecal/ileal intubation	Five incomplete colonoscopies, due to disease in four: obstructing benign (*n* = 2) or malignant (*n* = 1) stricturing and severe Crohn's colitis (*n* = 1)	99.8%
Adenoma detection rate	All colonoscopies	37.8%
	For eligible patients according to GESA guidelines^13^	43.8%
Sessile serrated lesion detection rate	All colonoscopies	23.2%
	For eligible patients according to GESA guidelines^13^	24.0%

FOBT, fecal occult blood test; GESA, Gastroenterological Society of Australia.

Ten patients (two males and eight females; median age 57.5 years) had AIN: condylomata acuminatum, *n* = 2; AIN1, *n* = 2; AIN2, *n* = 4; and AIN3, *n* = 2 (Table 2). These colonoscopic findings were new and incidental; they were not related to the indication for colonoscopy. Four of these patients had undergone colonoscopy between the previous 5 and 11 years. A past history of CIN was reported by four patients, all of whom were treated for CIN more than 10 years previously.

**Table 2:** Demographic data for patients with AIN and histopathology findings

Variables		*n*
Gender	Male	2
	Female	8
Age (years)	30–39	1
	40–49	2
	50–59	3
	60–69	3
	70–79	0
	≥80	1
AIN grade	Condyloma acuminatum	2
	AIN1	2
	AIN2	4
	AIN3	2

AIN, anal intraepithelial neoplasia.

The rate of detection of AIN per colonoscopy was 1/261 (95% CI 1/142–1/480) for all colonoscopies and 1/163 (95% CI 1/83–1/321) in women above 40 years of age. The 2‐year incidence rate of AIN was 1/253 (95% CI 1/137–1/464) for all patients. For women above 40 years of age, the 2‐year incidence rate of AIN was 1/157 (95% CI 1/79–1/307).

## Discussion

This is the first study to evaluate the rate of detection and incidence of AIN in a general population, without focusing on a high‐risk group. We found AIN in 1 of 253 adult patients receiving colonoscopy. For women aged over 40 years, AIN was found in 1 of 157. Although anal cancer is uncommon, the increasing incidence suggests there is benefit from opportunistically detecting AIN at colonoscopy. A total of 60–70% of patients with anal cancer present with advanced disease at diagnosis,^13^ making early detection or prevention important. This has particular relevance for the next 20–40 years as the unvaccinated population remains at risk of HPV infection.

Of those with AIN, the predominance of women over 40 years is an important observation. As mentioned before, and consistent with our results, previous studies have acknowledged the connection between CIN and AIN, reporting AIN in 10.4–17.4% of individuals with previous CIN.^7^, ^8^, ^9^, ^14^ Unlike the general population evaluated by us, these studies only enrolled patients with CIN. In those with previous CIN, AIN is more common over 35 years of age,^7^ which is important as colonoscopy becomes increasingly likely over 40 years of age.

A recently described pathophysiological mechanism of disease for women is that the genitals can act as a reservoir for HPV infection and subsequently cause anal infection. A 2016 Tasmanian study of women with previous HPV‐mediated gynecological neoplasia demonstrated a strong association between toileting habits and risk of anal neoplasia. Front‐to‐back wiping was associated with increased risk of anal neoplasia, while dabbing wiping demonstrated reduced risk of anal neoplasia.^10^ Considering common recommendations regarding front‐to‐back wiping to prevent urinary tract infection, a potential conflict in appropriate clinical direction arises.

Although our population sample is relatively small, we noted that four of our patients with AIN were treated for CIN at least 10 years previously. Natural history data regarding CIN and AIN progression to anal cancer are limited. One reported anal cancer developing between 4 and 16 years after HPV‐related gynecological neoplasia.^15^ Others report that AIN3 progresses to anal cancer at a rate of 9.5% over 5 years.^16^ With potentially lengthy intervals between CIN, AIN, and anal cancer, it is therefore appropriate to question the role of AIN screening in women with prior CIN. In light of the low overall prevalence of anal cancer, routine screening in healthy women without known risk factors is not recommended.^17^ Conversely, it has been recommended that high‐risk groups, including women with previous CIN, be questioned about AIN or anal cancer symptoms such as pain and bleeding and have digital rectal examinations.^17^ For women with CIN, it has been recommended that screening for AIN and anal cancer be performed within 5 years of diagnosis, with consideration of anal cytology and anoscopy.^17^ We believe that our report provides the basis for an expanded clinical algorithm that incorporates AIN screening with colonoscopic practice.

Our findings have implications for practicing endoscopists. First, previous CIN is an important risk factor for AIN, and it may help if endoscopists are made aware of a past history of CIN, however distant, at the time of colonoscopy. Second, the finding of AIN at colonoscopy requires a sympathetic patient consultation that, for women, includes an understanding that previous toileting habits may have been responsible for anal disease rather than anal receptive intercourse. Finally, as AIN is only accurately examined for endoscopically, diligent anorectal examination at colonoscopy allows for opportunistic AIN detection as an important adjunct to colorectal neoplasia detection.

Our report is necessarily limited in the amount of clinical data able to be communicated without the consent of all patients. For the endoscopist, we are not able to provide images of AIN detected but stress the importance of careful examination in both forward view and in retroflexion view where possible. Endoscopic appearances are often of nonulcerated, superficially elevated, plaque‐like, pitted lesions that may be subcentimeter in diameter and not easily palpable on digital examination, arising within the transitional zone between the squamous and columnar epithelium and which may extend both proximally and distally. It would have been beneficial to evaluate CIN prevalence within the total cohort of 1474 women, but it is not usual practice to ascertain this sensitive information prior to colonoscopy. While our research provides a possible target AIN detection rate for practicing endoscopists, further data in community and hospital‐based settings are desirable.

In summary, we provide a foundation to understanding the detection rate of AIN in individuals undergoing colonoscopy in a community‐based setting. Colonoscopy provides a useful opportunity to incidentally detect these premalignant lesions, particularly in women with a previous history of CIN and those over 40 years of age. Opportunistic AIN detection may mitigate the increasing incidence of anal cancer, especially in women, until longer‐term benefits of HPV vaccination come to fruition.
